# What Is the Common Ground for Modern Psychotherapy? A Discussion Paper Based on EACLIPT’s 1st Webinar

**DOI:** 10.32872/cpe.8403

**Published:** 2022-03-31

**Authors:** Stefan G. Hofmann, Jacques P. Barber, Paul Salkovskis, Bruce E. Wampold, Winfried Rief, Anne-Catherine I. Ewen, Leonora Nina Schäfer

**Affiliations:** 1 Department of Clinical Psychology and Psychotherapy, Philipps-University of Marburg, Marburg, Germany; 2 Gordon F. Derner School of Psychology, Adelphi University in Garden City, New York, NY, USA; 3 Department of Experimental Psychology, University of Oxford, Oxford, United Kingdom; 4 Counseling Psychology, University of Wisconsin – Madison, Madison, WI, USA

**Keywords:** psychotherapy, common ground, Process-Based Therapy, panel discussion, EACLIPT webinar

## Abstract

Psychotherapy as it is implemented today, can be seen as the composition of unconnected groups of practitioners and scientists pursuing different theories. The idea of finding a common “umbrella” for all evidence-based treatments in the field of psychotherapy is gaining more interest. Based on this background, experts in clinical psychology from various backgrounds led a fundamental discussion about modern psychotherapy and its basic mechanisms. Process-Based Therapy (PBT) was presented by Stefan Hofmann as a possible novel approach to clinical research and practice. In this article we present the different perspectives of the four panelists on PBT and in how far the model builds a common ground for different treatment approaches. Learning mechanisms and the therapeutic alliance were almost unanimously considered as indispensable factors in a global model of psychotherapy. In conclusion, the panelists emphasized a much-needed focus on characteristics and competencies of therapists themselves e.g., in communication, listening and empathy. These core competencies should be trained and promoted independently of the therapeutic approach.

## Highlights

- The formulation of a common ground of modern psychotherapy is needed that integrates all evidence-based psychological therapies.
- Process-Based Therapy was proposed as a new overarching concept that complements former general psychotherapy approaches.
- Basic mechanisms of psychotherapy were discussed that considered different psychotherapeutic approaches.
- Implications for education and training in psychological therapies should focus on a competence-based approach.

The European Association of Clinical Psychology and Psychological Treatment EACLIPT has the goal to promote and develop research in clinical psychology, its application and in psychological treatments and fostering the communication throughout the world. In this framework, a webinar has been organized by EACLIPT leading a theoretical discussion about “What is the common ground for modern psychotherapy?” with Stefan Hofmann, Jacques Barber, Bruce Wampold, and Paul Salkovskis as panelists, and chaired by Winfried Rief. The webinar was streamed live on the 16^th^ of November 2021, whereas already over 1.800 people watched the video (still online available on YouTube under the following link: https://www.youtube.com/watch?v=WffZx2lOlTs).

The background of organizing this expert panel was based on the idea of finding a common “umbrella” (EACLIPT, 2019; Rief, 2021), i.e. a common language, for all evidence-based treatments in the field of psychotherapy. The webinar was introduced with the following comments: Psychotherapy was developed from different roots, and many clinicians and scientists still consider psychotherapy as a collection of unconnected groups of theories and associated interventions. However, as long as psychotherapy is not considered as one academic and clinical field, progress and reciprocal stimulation of developments is seriously hampered. Goldfried labeled this stage as “prescientific”, and calls for search for a common ground, language and theory of psychotherapy, to develop one science and intervention model that could be used as overarching framework, before specifying into single approaches (Goldfried, 2020). Hofmann and Hayes believe that the evaluation of complete treatment packages (e.g., exposure for phobias) has reached its limit and needs more flexible, process-based, and problem-focused treatment planning, grounded in scientifically proven mechanisms of change (Hayes & Hofmann, 2018). Consequently, training of young clinical psychologists and psychotherapists may require a switch from a single traditional “school” of psychotherapy to a competence-based education that can integrate different, scientifically proven methods, derived from different backgrounds (Rief, 2021).

This article summarizes the main discussion points. A short introduction about the presented theoretical background of Process-Based Therapy (PBT) developed by Hayes and Hofmann (2018) is given, followed by the main statements about common ground theories of psychotherapy between the panelists.

## An Introduction to Process-Based Psychotherapy by Hofmann

Nowadays, clinical psychology based on the nomothetic approach focuses strongly on disorder categories and general treatment approaches instead of on the individual as well as on treatment change processes. Classification systems such as the ICD or DSM laid grounds to study various mental problems and provided effective alternatives to drug treatments. However, they are based on the latent disease model which cannot measure, quantify, or test these syndromes properly. Instead, syndrome clusters or disorders are an expression of symptoms based on a subjective report. Hofmann argued that clinical scientist should be more interested in the interrelationships of complaints and psychological variables, regardless of a possibly underlying latent disease model.

Within this context Hofmann referred to the complex network approach on clinical research of psychotherapy (Hofmann et al., 2016). This network perspective considers therapy as a highly complex process that involves a multitude of variables that typically form dynamic processes. To target these processes Hayes and Hofmann propose a transition from the nomothetic approach to an idiographic approach of theory-based and process-based therapy. Hofmann pointed out that PBT focuses on the biopsychosocial processes that should be targeted specifically for the given client and therapy goals to not only reduce symptoms but to enhance the client’s prosperity. Next, he referred to one of his own reviews which examined the most frequently validated mediators of psychosocial interventions. As a result, features such as self-efficacy, acceptance, expectations, psychological flexibility, coping skills etc. presented themselves as functionally important pathways of change, irrespective of being systemized into particular schools of psychotherapy.

Based on the idea to get away from a syndromic perspective, Hayes and Hofmann developed the so-called “*Extended Evolutionary Meta-Model”* (EEMM). As a meta model of adaptive change, the facilitation of clients’ competencies for adaption is targeted as a primary goal in PBT. This model is based on evolutionary theories assuming (mal-)adaptive change based on context-dependent variation, selection, and retention. To achieve a specific situational outcome, it is important to firstly be aware of various options (variation), secondly to select the most fitting one (selection) and thirdly to retain it (retention) for the given context, respectively. These change processes are expressed in interrelated dimensions of affect, cognition, attention, self, motivation, overt behavior, physiology and social background/ culture. By developing a whole problem network with the client called *‘grid’*, it is possible to identify maladaptation of the individual in the dimensions, respectively, and to help change it to an adaptive self-sustaining network. This concept opens the possibility to quantify and therefore predict (critical) psychological events (through e.g., critical slowing) such as psychotic breaks, suicide attempts or state of recovery.

In summary, the strategy of PBT is to depart from the latent disease model and embrace an idiographic and functional analytic approach. According to Hofmann, PBT emphasizes on flexibility and the widening of treatment goals from merely reducing negative affect towards positive affect to social connectedness, purpose, and quality of life.

## Discussion Points

The main discussion points are summarized in the following:

### Moving Away From a Syndrome-Based Approach: Is There an Additional Benefit of Process-Based Therapy as New Concept?

All panelists agreed on the current issues in clinical research and practice presented by Hofmann in his talk: Clinical psychology is too focused on the syndrome level, whereby the individual moves more and more in the background, especially in research. Psychotherapy is a more complex mechanism than just “reducing symptoms” or following treatment protocols. Salkovskis and Wampold pointed out, that these tedious issues are still leading to a constant formation of “new” therapy approaches which basically are still based on old concepts. These therapy approaches are not supposed to be disorder-specific but should rather focus on formulation and the client’s adaptation of that formulation as a mechanism of change. This covers helping the client to be less rigid and to formulate alternative interpretations of situations. By working in that collaboration therapy can help the person to learn how to operate in the world. Further, the novelty of the PBT approach was questioned as several process or contextual models of therapy were generated in clinical research over time. Other well-established concepts and therapy models were referenced which address similar therapeutic aspects as PBT such as epistemic trust, common factors model, the idea of flexibility or behavioral activation. Wampold and Salkovskis agreed with Hofmann that different approaches and interventions in therapy should be evaluated to improve the understanding of underlying processes. Hofmann added that the PBT approach is meant to provide broad guidelines from a wide length of therapeutic strategies to make therapy more individualized. From the PBT perspective therapy is a dynamic process of change. Through the complex network concept, it can be visualized and explained to the client as well as systematically adapted as goals change over time within the therapeutic context.

### Concept of Learning vs. Evolutionary Theory

The EEMM Model of PBT is based on an evolutionary perspective on adaptation. Building psychotherapy concepts on evolutionary processes was highly discussed between the panelists. This perspective was compared with learning principles as basic mechanism of therapy, whereas learning itself can be seen as an adaption process. It was pointed out that evolutionary theories are rather associated with long-term development processes on a group level whereas learning principles are possibly more applicable and comprehensible for patients on the individual level in a short-term context and therapeutic setting, respectively.

### Where to Find the Therapeutic Relationship in PBT?

The therapeutic relationship in the PBT-model can be found and considered in the social dimension. Salkovskis, Wampold, and Barber expressed the idea, to include it in a more salient way into the PBT-model, especially if PBT should present a ground for different psychotherapy traditions. As the need of an evidence-based grounding is crucial in psychotherapy, the therapeutic alliance should be included in experimental psychopathology research. It was proposed to go even further and to include the different aspects of a therapeutic relationship (e.g. communication; expectations about the therapeutic alliance; therapeutic alliance as corrective emotional experience). For psychodynamics this would be essential, as the therapist takes a much more active role in shaping and interpreting the therapist-patient relationship. Further on, computerized psychotherapy with no real therapeutic relationship was consulted. Even in this context, the therapeutic alliance could be seen as the expertise and authority presumed by the patient of the person they imagine behind the book or the program, whereas trust in the medium is discussed as crucial.

In agreement, Wampold pointed out that the therapeutic alliance in computer-assisted therapies is just as predictive as in face-to-face therapy.

### Inclusion of Different Therapy Training Approaches?

PBT aspires to be a therapy school- independent approach that integrates different therapy training approaches. In accordance with all panelists, the idea of finding a common language for different schools of therapy was welcomed. However, it was argued that although the combination in PBT of neurobiological aspects with psychology is admirable, important aspects of psychotherapy in general e.g., the ability to listen to somebody well are not instantly recognizable in this model. It was questioned if PBT rather creates a common language for cognitive-behavioral therapy (CBT) and not for all therapies. In response Hofmann pointed out that over the years ‘CBT’ has become a very broad term for a therapy school including constantly evolving therapy approaches and clinical strategies which do not necessarily represent the traditional image of CBT. It should therefore just be called “therapy”.

### Implications in Psychotherapy Training

According to Hofmann, one core issue is that therapists are mainly trained based on guidelines that are based on disorders, although therapy is a much more complex process than just reducing symptoms. The therapy school approach is too rigid. Related to this, the selection of therapist in training itself should be considered. Characteristics of a decent human being with the ability to empathize and collaborate with people should be looked out for. The importance of empirical grounding in training and the need of a permanently self-correcting system on the level of own therapy outcomes as well as on the service level was discussed. In this context the example of the IAPT *Improving Access for Psychological Treatment* by David Clark was proposed (Clark, 2018). Using feedback systems and informing about deviances from expected improvements, different measures can be taken such as proposing additional supervision or shifting the training. Furthermore, to evolve psychotherapy training, the question about what characterizes an effective therapist in delivering different treatments should be addressed. As a main criterion, therapists should be trained, regardless of what model they adopt, to do it effectively.

**Figure:**
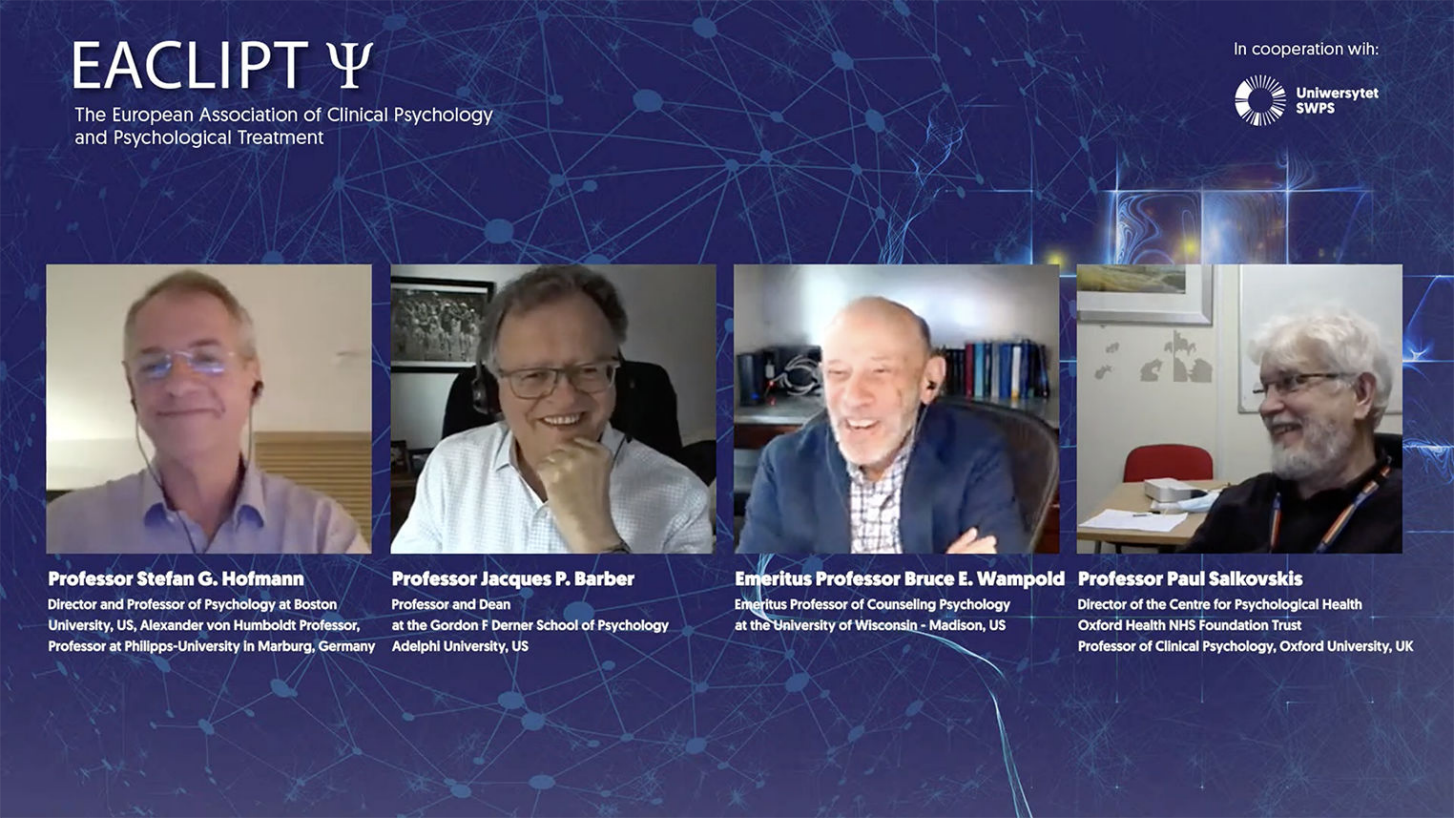


## Conclusion

The present paper summarizes the main discussion points between the panelists Jacques Barber, Stefan Hofmann, Paul Salkovskis, and Bruce Wampold on finding a common ground in psychotherapy within the context of the PBT model presented by S. Hofmann. The common agreement about getting away of a school-dependent system towards a more global approach considering school-independent factors became clear. PBT as a proposed modern therapy approach brought up different points of criticism and addition. The focus on more general evidence-based change processes is welcomed, but a better consideration of common factors was proposed, and the therapeutic alliance was specifically highlighted to be integrated. Moreover, the basic process of psychotherapy based on evolutionary theory in PBT was balanced against using the basic principles of learning. To conclude, the way to a common ground in psychotherapy is a highly important and well discussed topic, and when further perspectives are integrated, this can result in a dynamic and developing meta-model for psychotherapy.

## References

[r1] Clark, D. M. (2018). Realizing the mass public benefit of evidence-based psychological therapies: The IAPT program. In T. Widiger & T. D. Cannon (Eds.), *Annual Review of Clinical Psychology* (Vol. 14, pp. 159-183). 10.1146/annurev-clinpsy-050817-084833 PMC5942544 29350997

[r2] EACLIPT Task Force On “Competences of Clinical Psychologists”. (2019). Competences of clinical psychologists. Clinical Psychology in Europe, 1(2), e35551. 10.32872/cpe.v1i2.35551

[r3] Goldfried, M. R. (2020). The field of psychotherapy: Over 100 years old and still an infant science. Clinical Psychology in Europe, 2(1), e2753. 10.32872/cpe.v2i1.2753 PMC9645486 36397979

[r4] Hayes, S. C., & Hofmann, S. G. (2018). *Process-based CBT: The science and core clinical competencies of cognitive behavioral therapy*. New Harbinger Publications.

[r5] Hofmann, S. G., Curtiss, J., & McNally, R. J. (2016). A complex network perspective on clinical science. Perspectives on Psychological Science, 11(5), 597–605. 10.1177/1745691616639283 27694457 PMC5119747

[r6] Rief, W. (2021). Moving from tradition-based to competence-based psychotherapy. Evidence-Based Mental Health, 24, 115–120. 10.1136/ebmental-2020-300219 33468517 PMC8311107

